# Gastroesophageal Intussusception Treated With Combined Transoral Incisionless Fundoplication in a Patient With Systemic Sclerosis

**DOI:** 10.14309/crj.0000000000001632

**Published:** 2025-04-05

**Authors:** Areeka Memon, Nicholas Dugan, Thiruvengadam Muniraj, Monique E. Hinchcliff, Amir Masoud

**Affiliations:** 1 Section of Rheumatology, Allergy, & Immunology, Yale School of Medicine, New Haven, CT; 2 Department of General Surgery, Hartford Healthcare, Hartford, CT; 3 Section of Digestive Diseases, Yale School of Medicine, New Haven, CT; 4 Neurogastroenterology and Motility Center, Hartford Healthcare, Hartford, CT

**Keywords:** gastroesophageal intussusception, cTIF, diffuse cutaneous systemic sclerosis, transoral incisionless fundoplication

## Abstract

We present a case of a 49-year-old woman with diffuse cutaneous systemic sclerosis with refractory gastroesophageal reflux disease and dysphagia for pills and solid foods. Esophagogastroduodenoscopy revealed gastroesophageal intussusception. Despite several interventions including esophageal stent placement, dysphagia persisted. Owing to refractory severe symptoms, the patient underwent a laparoscopic hiatal hernia repair combined with endoscopic transoral incisionless fundoplication. The patient tolerated the intervention well and dysphagia resolved. Although an obscure persistent dysphagia in SSc patients, gastroesophageal intussusception should be considered. This case underscores the need for persistence in the diagnostic evaluation of gastrointestinal symptoms in patients with SSc and highlights the need for a multidisciplinary team care approach.

## INTRODUCTION

Systemic sclerosis (SSc) is a chronic autoimmune disease characterized by vasculopathy, fibrosis, and autoantibody production. Gastrointestinal (GI) manifestations, including gastroesophageal reflux disease (GERD) symptoms, are present in up to 90% of patients with SSc and significantly contribute to morbidity and mortality, accounting for approximately 12% of SSc-related deaths.^1–3^ Although transient small bowel intussusception has been documented in SSc, cases involving gastroesophageal (GE) intussusception are extremely rare. We present a case of GE intussusception in a patient with diffuse cutaneous SSc who experienced refractory GERD and dysphagia symptoms, was successfully treated with a combination of laparoscopic hiatal hernia repair and transoral incisionless fundoplication (cTIF).

## CASE REPORT

A 49-year-old woman with a 26-year history of dcSSc and polymyositis presented with persistent pill and solid food dysphagia and progressive GE reflux unresponsive to proton-pump inhibitors and H2 blockers. Her clinical history included Raynaud phenomenon, peptic stricture postdilation, and interstitial lung disease. Previous treatments included prednisone, methotrexate (discontinued due to interstitial lung disease), and mycophenolate mofetil.

Physical examination revealed microstomia, diffuse facial telangiectasias, and sclerodactyly with digital contractures. Laboratory tests showed a 1:320 speckled pattern of anti-nuclear antibodies but were negative for other specific autoantibodies including antitopoisomerase I (Scl-70), anticentromere, and anti-RNA polymerase III, as well as anti-RNP, SSA, SSB, dsDNA, and cyclic citrullinated peptide antibodies. Rheumatoid factor and creatine kinase were also within normal limits.

**Figure 1.**
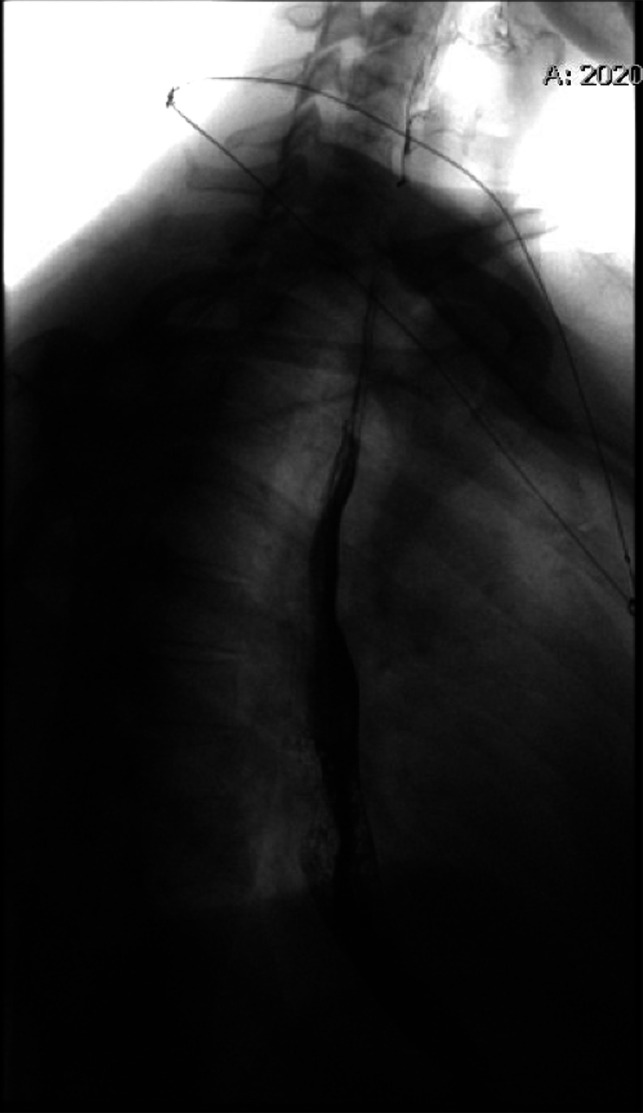
Esophagram revealing mild delayed clearance and mild stricture at the esophagogastric junction in association with irregularity at the esophagogastric junction.

**Figure 2.**
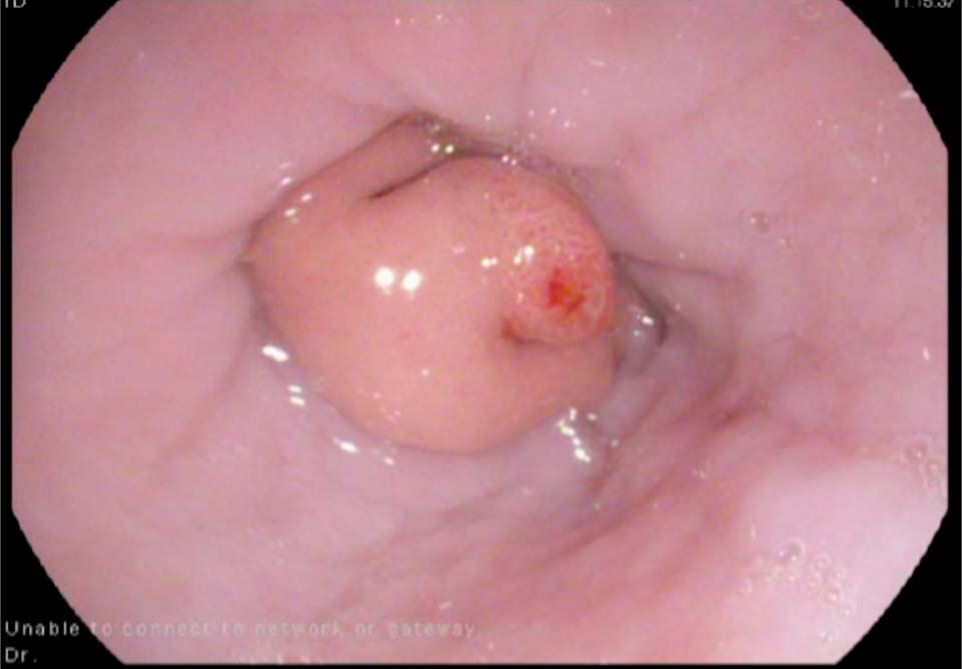
Photograph during esophagogastroduodenoscopy revealing gastric mucosa intussuscepting through the distal esophagus.

A barium esophagram showed delayed esophageal clearance, dysmotility, and GE reflux with an irregularity at the GE junction (Figure 1). High-resolution chest computed tomography revealed a patulous esophagus along with lower lobe predominant subpleural reticulations and bilateral lower lobe ground glass opacities. Despite treatment with alginate therapy and rabeprazole, symptoms persisted. Next, esophagogastroduodenoscopy (EGD) was performed that revealed an intermittently prolapsing and obstructing mass at the GE junction, suspected to be contributing to intussusception, and a moderate-sized hiatal hernia (Figure 2).

**Figure 3.**
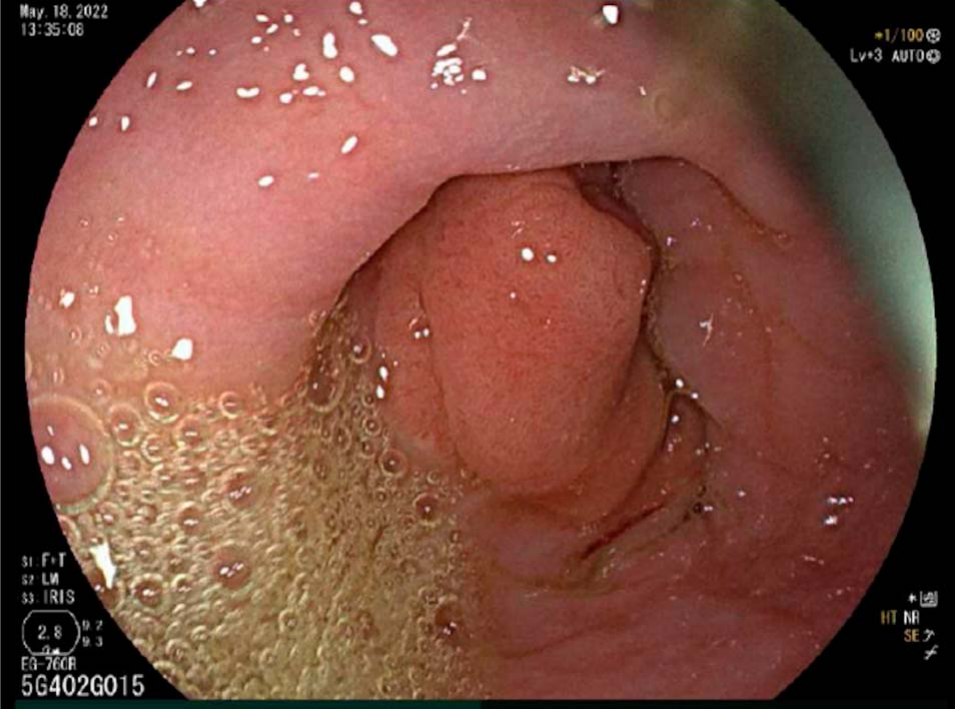
Repeat esophagogastroduodenoscopy following polyp removal and stent placement/removal revealing recurrent gastroesophageal intussusception.

It was hypothesized that the obstructing mass was the lead point for the GE intussusception, and thus, endoscopic ultrasound (EUS) was performed that confirmed the suspicion. During this procedure, an endoscopic mucosal resection of the polyp was performed. The pathology report noted 3 tissue pieces that measured on average 0.5 × 0.3 × 0.1 cm with foveal hyperplasia. A covered metal stent (AXIOS, Boston Scientific, Marlborough, MA) was placed across the GE junction to ensure patency and to prevent recurrence of intussusception. However, intractable pain necessitated stent removal within 2 weeks. Despite polypectomy, GERD and dysphagia continued, and a repeat EGD revealed recurrent GE intussusception into the lower third of the esophagus, a Hill Grade IV GE flap valve, and diffuse esophageal and GE junction dilation (Figure 3). Consequently, a cTIF was proposed.

**Figure 4.**
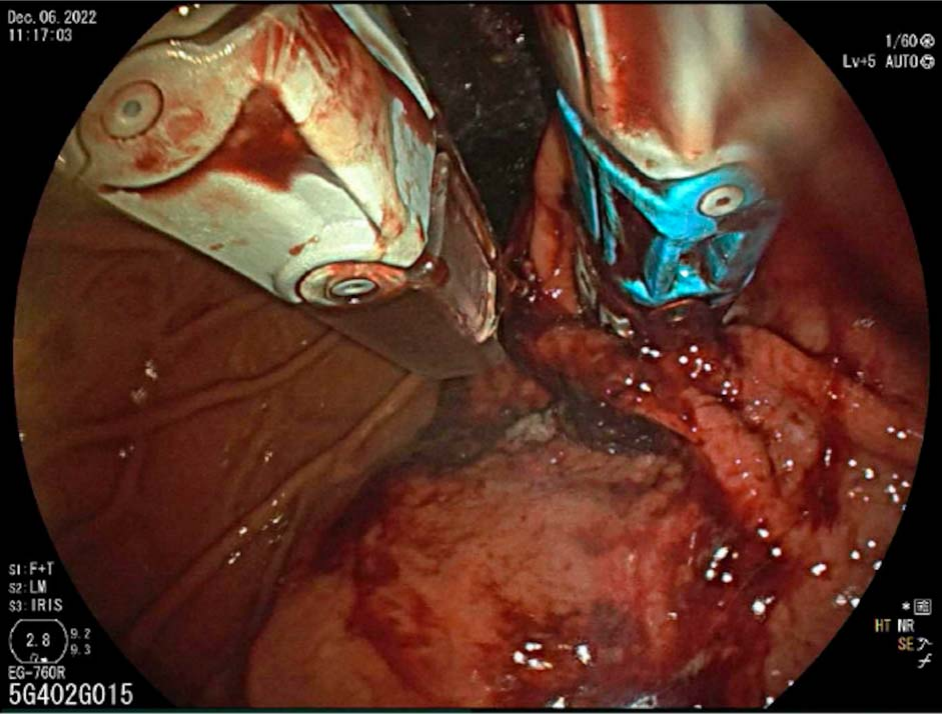
Gastroesophageal junction revealing lack of intussusception following combined with endoscopic transoral incisionless fundoplication procedure.

As a part of the cTIF, first, a laparoscopic hiatal hernia repair was performed. Next, endoscopic TIF was performed using the EsophyX Z device (EndoGastric Solutions, Redmond, WA) to reconstruct a GE barrier with placement of 20 fasteners (12 and 8 fasteners placed along the lesser and greater gastric curvatures, respectively) (Figure 4). A postprocedure endoscopy was performed and confirmed persistent resolution of GE intussusception. The patient has reported significant symptom improvement following the cTIF procedure that has been sustained up to the 3-year follow-up.

## DISCUSSION

We present a case of a woman living with diffuse cutaneous SSc who was diagnosed with a hiatal hernia, a gastric polyp, and GE intussusception. She underwent surgical polypectomy with placement of an esophageal stent that resulted in persistent pain necessitating stent removal. The patient ultimately underwent a hiatal hernia repair and TIF using the EsophyX Z device.^4^

GE intussusception, where the stomach invaginates into the esophagus, differs from a hiatal hernia, which involves the migration of abdominal contents through the diaphragmatic hiatus.^4–8^ This case is significant as it represents the first documented instance of GE intussusception in a patient with dcSSc, treated successfully with cTIF after conventional treatments failed. The pathogenesis of GE intussusception may include reverse gastric peristalsis, increased intra-abdominal pressure, redundancy of gastric mucosa, GE junction relaxation, and/or repeated regurgitation or vomiting.^5,6^ Congenital abnormalities such as malrotation with Ladd bands, superior mesenteric artery syndrome, and annular pancreas may underlie refractory symptoms.^3^ Hiatal hernias can exacerbate this condition by causing laxity in the phrenoesophageal ligament, allowing the GE junction to migrate above the diaphragm.^7^ The patient's combination of esophageal and GE junction dilation, a moderate-sized hiatal hernia, and gastroparesis likely contributed to her refractory symptoms.

Diagnostic imaging such as chest computed tomography and EGD can identify GE intussusception.^3,5^ EGD is important for diagnosis but can sometimes miss the pathology due to insufflation. Medications including proton-pump inhibitors, H2 blockers, and promotility agents were trialed in our patient to treat persistent GERD symptoms and dysphagia. Given reports that the frequency of GERD symptoms lessen and quality of life improves in patients with SSc, alginate therapy was also prescribed to no avail. Repeat EGD, EUS, and laparotomy were eventually performed underscoring the importance of persistence in working up patients with SSc-GI symptoms. Surgical and procedural interventions including gastropexy and fundoplication were considered, but concern for complications led to implementation of newer technologies that allow for transoral device implantation.^9,10^ Because the manufacturer warns against EsophyX Z device use in patients with esophageal dysmotility, fasteners were used to create a partial GE junction.^4^

We present a patient with long-standing dcSSc with refractory GERD symptoms and dysphagia who failed medical management. We demonstrate how transoral device implantation using the EsophyX Z device enabled the creation of a partial, yet effective GE junction, with symptom resolution and avoidance of complications with 3 years of follow-up.^9,10^ We underscore the importance of comprehensive GI evaluations to diagnose and treat the myriad SSc-GI manifestations that can occur (and that now include GE intussusception), a multidisciplinary team care approach to address refractory symptoms, and consideration for using newer TIF approaches including the EsophyX Z device system to treat SSc patients with refractory GERD symptoms and dysphagia.

## DISCLOSURES

Author contributions: A. Masoud, M. Hinchcliff, and A. Memon conceived the idea for the case report and wrote the manuscript. A. Masoud, N. Dugan, T. Muniraj, M. Hinchcliff, and A. Memon obtained clinical data for the patient. A. Masoud and N. Dugan obtained the images for the figures. All authors assisted in editing the manuscript. Amir Masoud is the article guarantor.

Financial disclosure: None of the authors declare any competing interests. NIH/NIAMS grant R01 AR073270 supported this work (MH).

Previous presentation: ACG 2023 EPoster Hall; 10/24/2023; Vancouver, British Columbia, Canada.

Informed consent was obtained for this case report.
